# Gadolinium Enhancement May Indicate a Condition at Risk of Developing Necrosis in Marchiafava–Bignami Disease: A Case Report and Literature Review

**DOI:** 10.3389/fnhum.2019.00079

**Published:** 2019-02-27

**Authors:** Zhiqin Wang, Jianfeng Wang, Fang Yi, Lin Zhou, Yafang Zhou

**Affiliations:** ^1^Department of Geriatrics, Xiangya Hospital, Central South University, Changsha, China; ^2^National Clinical Research Center for Geriatric Disorders, Xiangya Hospital, Central South University, Changsha, China

**Keywords:** Marchiafava–Bignami disease, alcoholism, MRI, gadolinium enhancement, necrosis, prognosis

## Abstract

Marchiafava–Bignami disease (MBD) is a rare condition characterized by demyelination, necrosis and atrophy of the corpus callosum (CC), and mainly associated with alcoholism. MBD may present with various clinical manifestations. Brain magnetic resonance imaging (MRI) scan is important in prompt diagnosis and treatment of MBD. Here we reported a case of MBD and reviewed literature about the usage of gadolinium-enhanced MRI in MBD. Gadolinium enhancement may indicate a condition at risk of developing necrosis. We therefore recommend a contrast-enhanced MRI study in severe alcoholics with suspected diagnosis of MBD.

## Background

Marchiafava–Bignami disease (MBD) is a rare condition characterized by demyelination, necrosis and atrophy of the corpus callosum (CC), and mainly associated with alcoholism, although some non-alcoholics have also presented with phenotypic and radiological findings that are typical of MBD (Hillbom et al., 2014). MBD may present with various clinical manifestations, including altered mental state, impaired walking, dysarthria, mutism, signs of disconnection syndrome, incontinence, seizures, and dementia (Hillbom et al., 2014; Fernandes et al., 2017). In the past, cases of MBD were diagnosed only at autopsy (Kawamura et al., 1985). The development of modern brain imaging techniques has allowed early detection of lesions suggesting MBD, and result in prompt diagnosis, treatment and better prognosis (Hillbom et al., 2014). Here we reported a case of MBD with gadolinium accumulation in the lesion, and reviewed literature about the usage of gadolinium-enhanced Magnetic resonance imaging (MRI) in MBD.

## Case Presentation

The patient was a 43-year-old man admitted to our hospital with 5 days history of slurred speech, unsteady gait, altered mental state, seizures and incontinence. The patient had been consuming an average of 250 mL of spirit (Chinese liquor, ≥ 52% v/v) per day for the last 25 years. Upon admission, the patient was in coma with a Glasgow Coma Scale (GCS) of 9. Physical examination showed normal pupillary size and reaction. Muscle tone and tendon reflexes were normal. Plantar cutaneous reflexes exhibited bilateral flexion.

The baselines CBC were within normal limits except for mild anemia (119 g/L). Electrolytes (sodium, potassium, magnesium, and phosphate), calcium, chloridion, albumin levels, creatinine, urea, blood lipids, blood glucose, C reactive protein and thyroid were normal. ELISA for HIV and syphilis were negative. Testing for antibodies and antigens of hepatitis B and C were all negative, except for positive HBsAb. Baseline vitamin levels were not obtained. Cerebrospinal fluid showed a slightly increased protein level of 0.64 g/L, with normal nucleated cell count, glucose, chloridion and negative viral IgM. Gram’s stain, acid-fast stain and India ink stain for cerebrospinal fluid were all negative.

**FIGURE 1:**
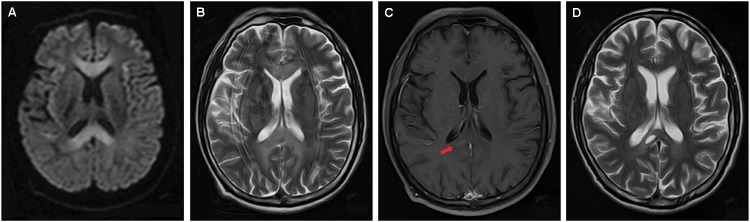
Axial images of brain MRI performed 7 days **(A–C)** and 22 days (D) after onset. MRI performed 7 days after onset revealed symmetrical and bilateral hyperintense lesions on diffusion-weighted imaging (DWI) **(A)**, and T2-weighted imaging (B) throughout the entire corpus callosum (CC). Extracallosal lesions could also be seen **(A,B)**. T2-weighted imaging had a considerable amount of distortion due to motion because the patient couldn’t cooperate well **(B)**. Gadolinium enhancement could be seen in part of splenium **(C, arrow)**. 22 days after onset, follow-up MRI showed only mild remaining of the formerly impressively hyperintensity on D2-weighted imaging, and the formation of necrosis in the splenium **(D)**.

**FIGURE 2:**
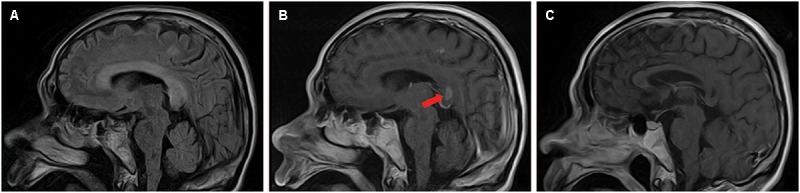
Sagittal images of brain MRI performed 7 days **(A,B)** and 22 days **(C)** after onset. MRI performed 7 days after onset revealed hyperintense lesions on T2-weighted imaging throughout the entire CC and in some extracallosal regions **(A)**. Lesions that were enhanced by gadolinium could be seen in the splenium (arrow) and some extracallosal regions **(B)**. Follow-up head MRI performed 22 days after onset showed necrosis without enhancement had occurred in the formerly gadolinium-enhanced lesions **(C)**.

Magnetic resonance imaging (MRI) was performed 7 days after onset on a 1.5 T magnet (Toshiba, 1.5 T, EXCELART vantage MRT-1503 Atla-Basic) with the following parameters: proton density-weighted imaging (PDWI): TR/TE of 1400 ms/15 ms; T2WI: TR/TE of 4300 ms/105 ms, slice thickness 5 mm, interslice gap of 1.5 mm; DWI: TR/TE of 5300 ms/100 ms, field of view was 240 mm, two b values were acquired (0 and 1000 s/mm^2^), slice thickness was 5 mm, and interslice gap was 1.5 mm; fast fluid attenuated inversion recovery (FLAIR) imaging: TR/TE was 8000 ms/105 ms, field of view was 240 mm, TI was 2200 ms. Postcontrast PD-weighted (TR, 1600 ms; TE, 15 ms) images were acquired after intravenous administration of 0.2 mL/kg body weight of gadopentetate dimeglumine at a rate of 2 mL/s. The MRI revealed symmetrical and bilateral hyperintense lesions throughout the entire CC, and in scattered parts of bilateral hemispheric white matter and cortex, visualized on diffusion-weighted imaging (DWI) (Figure 1A), T2-weighted (Figure 1B), and fluid attenuated inversion recovery sequence (FLAIR) (Figure 2A) imaging. Lesions that were enhanced by gadolinium could be seen in the splenium and some extracallosal regions (Figure 1C, 2B).

A diagnosis of MBD was made. The patient was treated with thiamine (100 mg/d) and mecobalamin (500 μg/d) intramuscular. Three weeks after symptoms onset there was significant improvement. The patient’s consciousness was improved with a GCS of 13. He was able to move all limbs, have simple conversation and control urination and defecation.

Follow-up head MRI was performed 22 days after onset on a 1.5 T magnet (Toshiba, 1.5 T, EXCELART vantage MRT-1503 Atla-Basic) with the following parameters: PD-weighted imaging (PDWI): TR/TE of 1550 ms/15 ms; T2WI: TR/TE of 4300 ms/105 ms, slice thickness 5 mm, interslice gap of 1.5 mm; fast fluid attenuated inversion recovery (FLAIR) imaging: TR/TE was 8000 ms/105 ms, field of view was 240 mm, TI was 2200 ms. Postcontrast PD-weighted (TR, 1476 ms; TE, 15 ms) images were acquired after intravenous administration of 0.2 mL/kg body weight of gadobenate dimeglumine at a rate of 2 mL/s. Corresponded to the clinical improvement, follow-up head MRI performed 22 days after onset showed only mild remaining of the formerly impressively hyperintensity on T2-weighted imaging (Figure 1D). Intriguingly, necrosis without enhancement had occurred in the formerly gadolinium-enhanced lesion (Figure 1D, 2C).

## Discussion

Marchiafava–Bignami disease is a rare condition. Wilson et al. (2017) reviewed 174 cases of callosal lesions with restricted diffusion. Among them, 47% were vascular and 53% were nonvascular. The most common nonvascular etiologies were trauma (44%), tumor (22%), and demyelination (15%). None of them was identified as MBD. In MBD, hyperintensity on DWI may be caused by reversible myelin vacuolation or intramyelinic edema (Tung et al., 2010). Intriguingly, in our case, only part of hyperintensity lesion visualized on DWI appeared to have gadolinium enhancement. This region also corresponded to necrosis on follow-up MRI. Nevertheless, the significance of gadolinium enhancement has not been systemically evaluated in MBD.

To our knowledge, only 10 cases of MBD had been reported with both initial gadolinium-enhanced MRI performed in acute stage and repeated MRI (either non-contrast or contrast-enhanced) performed in subacute or chronic stage since 1985 (Table 1). 2/10 showed enhancement of CC lesions on the initial MRI. Both two cases showed necrosis of formerly gadolinium-enhanced lesions on repeated MRI. Interestingly, we found another case, in which contrast-enhanced CT was performed in the initial evaluation instead of MRI. Contrast enhancement was seen in the splenium. 21 days after the onset, MRI scan also showed the necrosis of the initial enhanced lesion (Caparros-Lefebvre et al., 1994).

**Table 1:** Neuroradiologic features in 10 reviewed cases of MBD.

Author/year (Reference)	Initial postcontrast MRI		Repeated MRI		Glasgow outcome score (GOS)
	Day after onset	Enhancement	Day after onset	Necrosis	
Alghamdi et al., 2018	xa	Yes	About 3 months	Yes	Dead
Kilinc et al., 2015	6	No	16	No	Normal
Wenz et al., 2014	1	No	4	No	Minimal disability
Consoli et al., 2014	2	No	12	Yes	Normal
Sehgal et al., 2013	5	No	3 months	No	Normal
Tung et al., 2010	5	No	17^∗^	No	Normal
Caulo et al., 2009	1	No	4 months	No	Moderate disability
Bano et al., 2009	xa	No	About 3 weeks	No	Normal
Hlaihel et al., 2005	4	No	30	No	Moderate disability
Caparros-Lefebvre et al., 1994	9	Yes	3 weeks	Yes	Moderate disability
Caparros-Lefebvre et al., 1994	2	Yes^∗∗^	23 (MRI)	Yes	Moderate disability


*Xa, not specified, acute stage. ^∗^another follow-up MRI 3 months after onset failed to showed necrosis. ^∗∗^contrast-enhanced CT was performed in the initial evaluation instead of MRI.*

8/10 cases showed no enhancement of CC lesion on initial MRI. Among these 8 cases, no necrosis was reported in 7 cases on follow-up MRI preformed in subacute or chronic stage (Table 1). The only exception was one case reported by Consoli et al. (2014).

Outcome of these 10 cases was assessed by Glasgow Outcome Score (GOS). 5 of 8 cases without gadolinium enhancement on initial MRI recovered completely after prompt treatment. No death was reported. For two patients with gadolinium enhancement on MRI, one was dead and the other one had moderate disability.

Gadolinium enhancement is related to the break-down of the blood-brain-barrier (Caparros-Lefebvre et al., 1994). By reviewing literature, it is likely that gadolinium enhancement during acute stage of MBD indicates severe damage of lesion and may lead to necrosis in the chronic stage and is associated with poorer clinical outcome. Due to the limitation of cases reported, more clinical data is needed to draw a solid conclusion. We therefore recommend a contrast-enhanced MRI scan in severe alcoholics with suspected diagnosis of MBD.

## Conclusion

We reported a MBD case and evaluated the usage of gadolinium-enhanced MRI in MBD. Gadolinium enhancement may indicate a condition at risk of developing necrosis. We therefore recommend a contrast-enhanced MRI study in severe alcoholics with suspected diagnosis of MBD.

## Ethics Statement

All clinical data in this case report were either provided by the patient and his spouse or collected by our team’s members with the consent of them. There was no additional invasive test or experimental drugs used out of order for the patient. A written informed consent was obtained from the patient and his spouse for the participation in the study and the publication of this report. The case report is exempt from institutional review board approval.

## Author Contributions

ZW, JW, FY, and YZ acquired the clinical data, reviewed the literature, and drafted the manuscript. LZ and YZ designed the study, oversaw data acquisition, supervised the initial drafting, critically revised the manuscript. ZW and YZ reviewed the literature and revised the manuscript.

## Conflict of Interest Statement

The authors declare that the research was conducted in the absence of any commercial or financial relationships that could be construed as a potential conflict of interest.
